# Evaluation of dentinogenesis inducer biomaterials: an *in vivo* study

**DOI:** 10.1590/1678-7757-2019-0023

**Published:** 2019-11-15

**Authors:** Anabela B. Paula, Mafalda Laranjo, Carlos-Miguel Marto, Siri Paulo, Ana M. Abrantes, Bruno Fernandes, João Casalta-Lopes, Manuel Marques-Ferreira, Maria Filomena Botelho, Eunice Carrilho

**Affiliations:** 1 Universidade de Coimbra, Faculdade de Medicina, Instituto de Prática Clínica Integrada, Coimbra, Portugal.; 2 Universidade de Coimbra, Faculdade de Medicina, Instituto de Biofísica, Coimbra, Portugal; 3 Universidade de Coimbra, Faculdade de Medicina, Instituto de Pesquisa Clínica e Biomédica, area of Environment Genetics and Oncobiology (CIMAGO), Coimbra, Portugal; 4 Universidade de Coimbra, CNC.IBILI, Coimbra, Portugal; 5 Universidade de Coimbra, Faculdade de Medicina, Instituto de Patologia Experimental, Coimbra, Portugal; 6 Centro Hospitalar e Universitário do Porto, Departamento de Patologia, Porto, Portugal; 7 Coimbra University Hospital Center, Radiation Oncology Department, Coimbra, Portugal

**Keywords:** Biomaterials, Dentinogenesis, Dental pulp, Odontoblast, Pulp capping

## Abstract

When exposure of the pulp to external environment occurs, reparative dentinogenesis can be induced by direct pulp capping to maintain pulp tissue vitality and function. These clinical situations require the use of materials that induce dentin repair and, subsequently, formation of a mineralized tissue. Objective: This work aims to assess the effect of tricalcium silicate cements and mineral trioxide aggregate cements, including repairing dentin formation and inflammatory reactions over time after pulp exposure in Wistar rats. Methodology: These two biomaterials were compared with positive control groups (open cavity with pulp tissue exposure) and negative control groups (no intervention). The evaluations were performed in three stages; three, seven and twenty-one days, and consisted of an imaging (nuclear medicine) and histological evaluation (H&E staining, immunohistochemistry and Alizarin Red S). Results: The therapeutic effect of these biomaterials was confirmed. Nuclear medicine evaluation demonstrated that the uptake of ^99m^Tc-Hydroxymethylene diphosphonate (HMDP) showed no significant differences between the different experimental groups and the control, revealing the non-occurrence of differences in the phosphocalcium metabolism. The histological study demonstrated that in mineral trioxide aggregate therapies, the presence of moderate inflammatory infiltration was found after three days, decreasing during follow-ups. The formation of mineralized tissue was only verified at 21 days of follow-up. The tricalcium silicate therapies demonstrated the presence of a slight inflammatory infiltration on the third day, increasing throughout the follow-up. The formation of mineralized tissue was observed in the seventh follow-up day, increasing over time. Conclusions: The mineral trioxide aggregate (WhiteProRoot^®^MTA) and tricalcium silicate (Biodentine™) present slight and reversible inflammatory signs in the pulp tissue, with the formation of mineralized tissue. However, the exacerbated induction of mineralized tissue formation with the tricalcium silicate biomaterial may lead to the formation of pulp calcifications

## Introduction

Some clinical situations such as deep cavities, severe crown trauma, and iatrogenic situations may lead to pulp tissue exposure of the external oral environment.

Dental pulp has a natural potential for tissue repair, which leads to the formation of reparative dentin. It has been well documented that dental pulp can form a hard tissue barrier (dentin bridge) after directly capping the pulp or pulpotomy. During reparative dentinogenesis, the original odontoblasts at the site of exposure are destroyed and replaced by newly differentiated odontoblast-like cells. This process involves progenitor cells migration to the lesion site and subsequent proliferation and differentiation of these cells into odontoblasts. Thus, when the dental pulp tissue is exposed to the external environment, reparative dentinogenesis can be induced by pulp capping, to maintain pulp tissue vitality and function.^1^^–^^4^

Direct pulp capping consists of biocompatible materials and bio-conductors application in the exposure zone of the tissue in order to seal the communication, acting as a barrier, and at the same time protecting the pulp complex and consequently preserving its vitality. The fundamental characteristics of these materials are their biocompatibility, which includes antibacterial capacity and properties that induce tissue healing; cytocompatibility, and ability to seal the lesion.^5^ Several authors report that mineralized tissue induction formation by pulp cells is the main function of the biomaterial used for this type of therapy.^5^^,^^6^ The direct pulp capping regenerative treatment objective is the induction of odontoblast-like cells differentiation and consequently the formation of tertiary dentin in the exposure area with tissue structure reorganization. Some characteristics are common to most biomaterials indicated for therapeutics, protecting the pulp tissue vitality and which induce reparative dentin. These characteristics include high pH, antimicrobial activity and release of calcium ions.^4^^,^^7^^–^^10^

Calcium hydroxide is the most popular agent for direct and indirect pulpal capping and for maintaining pulp vitality due to its ability to release hydroxyl and calcium ions after dissolution. This biomaterial is the most studied and documented in several cellular, animal, and clinical studies and presents satisfactory results with success rates up to 80%, which is considered gold standard.^6^^,^^11^^,^^12^ However, there are some disadvantages such as poor adhesion to dentin, high solubility, and mechanical instability and consequent dissolution of the dentin bridged material with multiple tunnel defects.^11^^,^^13^^,^^14^

Developed in the 1990s, mineral trioxide-based cements received great attention, initially as a retrograde filling material, and its indications were expanded as a material for direct pulp capping. Although there are clinical evidence of the calcium hydroxide-based cements and mineral trioxide aggregates use in direct pulp capping therapies with satisfactory results, the best clinical performance in relation to each other is inconclusive.^6^^,^^15^ Recently, tricalcium silicate cements, such as Biodentine^™^, have emerged. This biomaterial has a calcium hydroxide-base and characteristics such as mineral aggregate trioxide cements but with tightening times substantially more suitable for their application and other clinical advantages.^14^^,^^16^^–^^18^ There are few studies about this material, and it was critical to compare its behavior with others.

The existing gap in the microenvironment influence, especially vascularization, internal fluids, and endogenous growth factors, without which it is not possible to answer the research question, led to the necessity to carry out the studies *in vivo*. Several studies have demonstrated that pulp tissue healing from a rat molar after pulp capping is histologically comparable to what occurs in humans and other animal species.

Thus, the *in vivo* animal model study aimed to evaluate the bioactive effect of Biodentine^™^, namely the formation of reparative dentin and inflammatory effects reduction over time after pulpal exposure. For this evaluation, the biomaterial was compared with the gold standard material for this therapy, an aggregated trioxide mineral based cement, WhiteProRoot^®^ MTA.

## Methodology

This work was approved by the Research Ethics Committee of the Faculty of Medicine of the University of Coimbra, respecting all legal provisions in force, after approval by ORBEA (Organ Responsible for Animal Welfare) and DGAV (Directorate-General for Food and Veterinary Medicine) – technical advice 7/2015.

### Sample calculation

Sample size was calculated using G*Power version 3.1.9.4 with an α level of 5% and 80% of power.

### Biomaterial treatment

For this study, 45 male Wistar Han healthy rats between 12 and 14 weeks old, with a mean mass of 205±30.91 grams, originating from the university medical school vivarium, were used. During the experimental period, the animals were kept in laboratory conditions, in accordance with the legislation in force (Decree-Law no. 113/2013 of August 7, 2013, transposing Directive 2010/63/EU of the European Parliament and of the Council of 22 September 2010). All animals were observed daily. During the experiment all animals were submitted to normal maintenance and nutrition, with an ambient temperature of 22°C and 12-h light-dark cycle.

**Figure 1:**
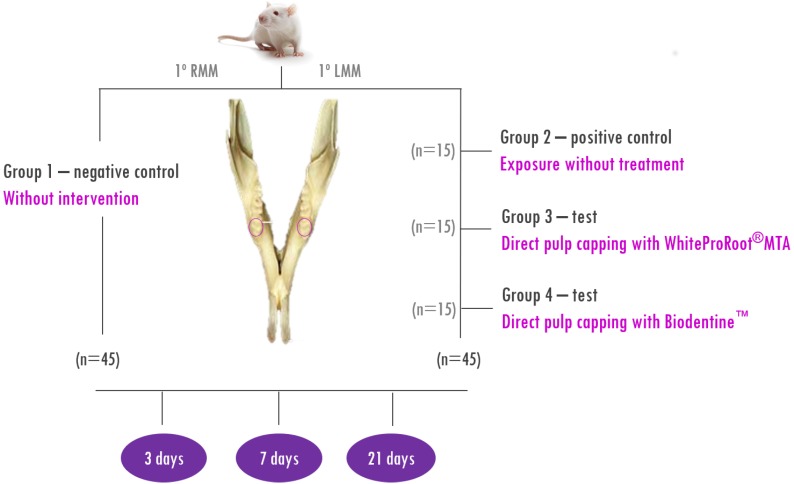
Split-mouth study design (RMM – right mandibular molar; LMM – left mandibular molar; no. – number of animals)

The 45 Wistar Han rats used were randomly divided into four groups in a split-mouth study design: two control groups, and two test groups as shown in Figure 1. All animals handled were weighed and anesthetized with 77% ketamine (25 mg/kg) and 33% chlorpromazine (25 to 40 mg/kg) intraperitoneally.

In the right first mandibular molars no intervention was performed in the negative control (Group 1). After disinfection of the teeth dental surface with 0.12% chlorhexidine, the pulp exposures were performed in the mandibular left first molars with a spherical diamond drill bit 008 in multiplier contra-angle and finished with the aid of a K 10 file. The cavity was irrigated with 2% sodium hypochlorite (DentaFlux; Madrid, Spain) followed by 2% chlorhexidine (Corsodyl^®^ Original Mouthwash, GlaxoSmithKline; Brentford, United Kingdom), and hemostasis was performed with a sterilized cotton ball. In the positive control group, Group 2, pulp exposure was performed without pulp capping treatment, and cavities were restored with a Ketac^™^ Fil Plus Aplicap^™^ (3M ESPE; St. Paul, Minnesota, USA) glass ionomer cement. In both test groups, the WhiteProRoot^®^ MTA (Group 3) and Biodentine^™^ (Group 4) were used to cap the exposed pulp tissue, followed by glass ionomer restoration with Ketac^™^ Fil Plus Aplicap^™^. Both biomaterials were manipulated according to the manufacturers’ instructions. After the intervention, all animals were monitored 4 times a day with wet food placed in the cage. They received analgesia with ibuprofen (10 mg/kg every 8 hours in the 24 hours after intervention).

In total 15 animals, corresponding to five animals from each intervention group, were killed at three, seven and twenty-one days after the surgical procedure, by anesthetic overdose.

### Acquisition of molecular imaging by Nuclear Medicine

In this study, the animals were anesthetized at three, seven and twenty-one days after treatment with the biomaterials with ketamine 50 mg/ml (Ketalar^®^, Parke-Davis; Barcelona, Spain) and received the administration of hydroxymethylene diphosphonate (^99m^Tc-HMDP). After 90 minutes of the radiopharmaceutical (9.2±0.31 MBq) intravenous administration, the animals were euthanized, and the jaws subsequently removed. In all specimens, a static image with a 256x256 Zoom1 matrix was acquired for two minutes.

After image acquisition, processing was performed at the Xeleris processing station by regions of interest (ROI) drawing. From the region of interest drawn for each hemimandible, maximum values count in the operated hemimandible (Groups 2, 3 and 4) and the mean counts in the contralateral hemimandible (Group 1) were obtained, in order to calculate the ratio between operated hemimandible and contralateral hemimandible.

The objective of ^99m^Tc-HMDP administration is to evaluate adsorption of the hydroxyapatite crystal on the surface and to correlate this information with the formation of new mineralized tissue.

### Histological analysis

Immediately after euthanasia, spleen, liver, lung, and mandible samples were collected at necropsy and processed for histological analysis. Hemimandibular samples were also conditioned, cataloged and fixed in 10% buffered neutral formaldehyde for seven days, washed in running water and then decalcified with the ethylenediaminetetraacetic acid (EDTA) solution in increasing concentrations at 4°C. Samples dehydration was then carried out, in an ascending battery of alcohols and later inclusion in paraffin.^19^^–^^21^

Longitudinal cuts were performed in the mesiodistal direction, approximately 5 μm thick and at 70 μm intervals, corresponding to the exposure region and pulp capping. The staining plates were prepared with three different techniques: hematoxylin-eosin (H&E), immunohistochemistry for the detection of Dentin SialoProtein (DSP), and Alizarin Red S. H&E staining was performed on the samples due to its simplicity and ability to enable visualization of many different tissue structures. The immunohistochemical staining technique was used to observe DSP expression. Sections were dewaxed with xylol, hydrated in a decreasing series of ethanol concentrations, and washed with PBS before being subjected to the primary antibody (DSP – M-20 – Antibody, Santa Cruz Biotechnology, Inc; Heidelberg, Germany, 1:100) and followed by the secondary antibody (Polyclonal Rabbit Anti-goat immunoglobulins/HRP, Dako; Glostrup, Denmark, 1:100). The antibody-antigen complex was detected by activation of peroxidase (Substrate Buffer; Dako, Glostrup, Denmark) and chromogen (DAB^+^ Chromogen, Dako; Glostrup, Denmark). The counterstaining was performed with hematoxylin. The objective of this staining was the mineralization assessment by DSP production after the treatments with the biomaterials.^19^^,^^22^^,^^23^ A third staining technique was also performed to detect calcium deposits formed by staining with Alizarin Red S. The solution Alizarin Red S at a 40 mM concentration was used. For this solution, the pH value is critical and should be between 4.1 and 4.3. The 4.2 pH was adjusted with NH_4_OH and HCl.^24^^–^^28^ The samples were stained with an Alizarin Red staining solution for 20 minutes at 37°C.^29^^,^^30^

**Figure 2:**
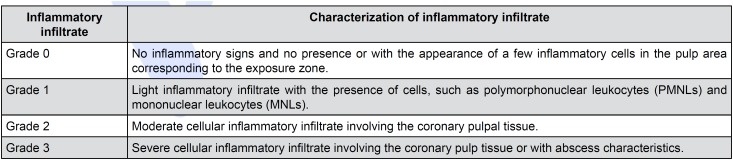
Classification of cellular inflammatory infiltrate

**Figure 3:**
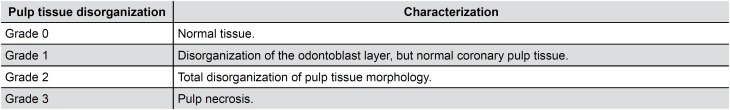
Classification of pulp tissue disorganization

**Figure 4:**
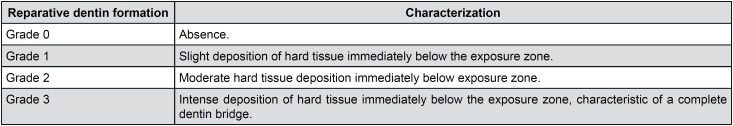
Classification of reparative dentin formation

Thus, the qualitative assessment based on the images obtained by H&E staining and DSP expression was based on the classification of Figures 2, 3 and 4, according to the modified version of ISO 10993 and 7405.^20^^,^^31^

The objective of H&E staining was to assess the inflammatory infiltrate, for the degree and extension to associated cell identification; pulp tissue disorganization, from the normal tissue to the total disorganization of the tissue with necrosis morphology; and the assessment of restorative dentin formation, from the absence of a dentin bridge to its complete edification. The objective of Alizarin Red S staining was to enable the assessment of mineralization by the formation of calcium deposits after treatment with the biomaterials.

Microscopic observation of all histological sections stained with the three techniques was performed using light microscopy on a Nikon Eclipse NI microscope. The photographs were obtained from the Nikon OS-Fi2 camera coupled to the microscope and later the images were captured and analyzed using the NIS-Elements D software. Photographs with 40x magnification, 100x magnification, and 200x magnification were obtained.

### Statistical analysis

Statistical analysis was performed using IBM SPSS software v.2 (IB5M Corporation, Armonk, NY, USA). Normal distribution was assessed by a Shapiro-Wilk test. According to the results Mann–Whitney *U* test was performed. Comparing several conditions, ANOVA parametric test or Kruskal–Wallis nonparametric test was used when the differences were considered statistically significant for p<0.05.

## Results

During the research period, the animals showed a healthy appearance with motor activity and normal breathing.

### Nuclear medicine assessment using ^99m^Tc-HMDP

**Figure 5:**
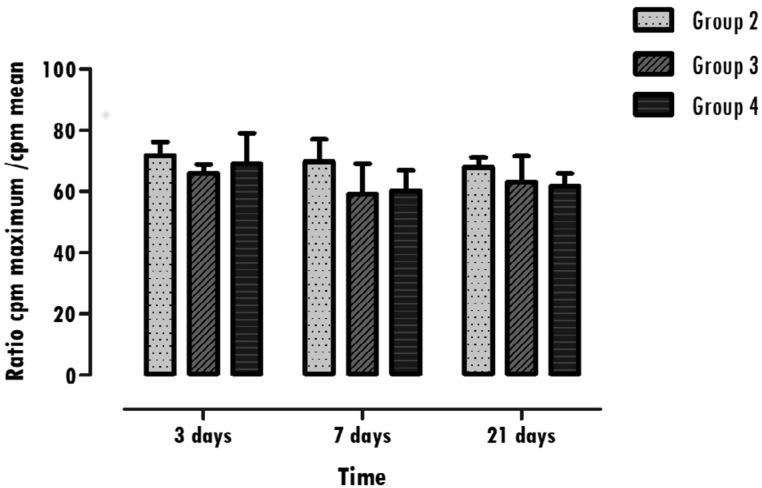
Representative graphic of the results obtained with the molecular image after 99mTc-HMDP administration. The values represent the mean and standard deviation of the ratio obtained between the operated hemimandible maximum counts and contralateral hemimandible mean counts at three, seven and twenty-one days in each group with different biomaterials, Groups 2, 3, and 4

All samples collected from the studied animals were included in the functional study. The results obtained after ^99m^Tc-HMDP administration revealed no statistically significant differences (p>0.05), when comparing each group in the periods of three, seven and twenty-one days, as can be seen in Figure 5. When the differences between those groups with different materials were assessed at each stage, it was found that there are no statistically significant differences (p>0.05).

### Biomaterials therapy analysis

All samples collected from the studied animals were included in the histological analysis. The choice of images was random.

**Figure 6:**
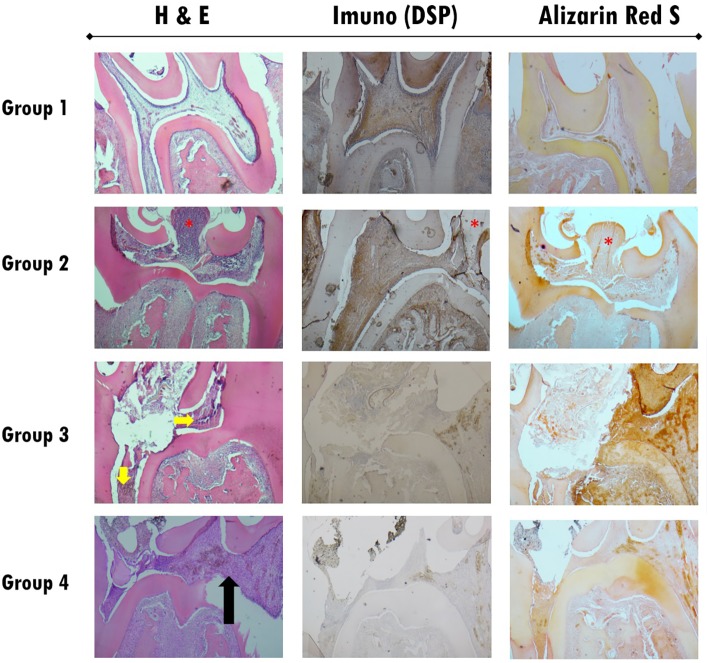
Histological section images of all study groups at three days follow-up, stained with H&E (left column), by immunohistochemistry for DSP (central column) and Alizarin Red S (right column). All images were obtained with 40x magnification. Group 2 shows a pulp polyp formation in the exposure zone without treatment represented by a red asterisk. In Group 3 the presence of the biomaterial used in the extensive exposure zone can be observed, with the presence of intense inflammatory infiltrate (Grade 2) and pulp tissue total disorganization (Grade 2) represented by yellow arrows. Immunohistochemistry images and staining with Alizarin Red S do not show alterations in the presence of DSP or calcium deposits (Grade 0). In Group 4, a slight inflammatory infiltrates (Grade 0) is observed next to the exposure zone (black arrow)

Normal pulp morphology as the baseline can be observed in Group 1 within all stages and all stain techniques. In Figures 6 and 11, the histological results can be observed after biomaterials therapies at three, seven and twenty-one days of follow-up.

**Figure 7:**
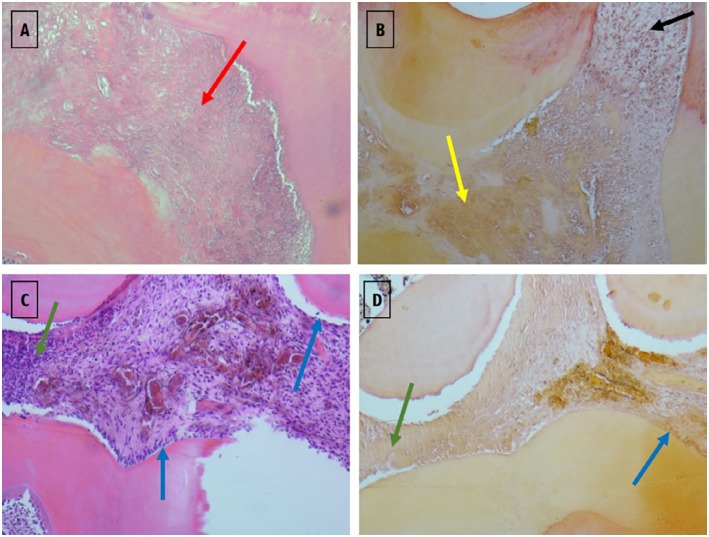
Images of histological sections in groups 3 (images A and B) and 4 (images C and D), in which therapies with WhiteProRoot^®^ MTA and Biodentine™ were performed, at 3 days of follow-up. Images A (Group 3) and C (Group 4) were stained with H&E and obtained with 100x magnification. The B (Group 3) and D (Group 4) images were stained with Alizarin Red S and obtained with 100x magnification. Red arrow – disorganized pulpal tissue with increased loose connective tissue; yellow arrow – disorganized pulp tissue with the presence of calcium deposits; black arrow – area corresponding to normal pulp tissue organization; green arrow – disorganized pulpal tissue with slight inflammatory infiltrates; blue arrow – odontoblast monolayer integrate

On day three (Figures 6 and 7), a italicstantial amount of inflammatory cell infiltration could be detected in the positive group (Group 2), and in both test groups — WhiteProRoot^®^MTA group (Group 3) and Biodentine^™^ group (Group 4) — but it was more visible in the positive group with the pulp polyp formation (Figure 6). In group 3, the H&E and staining with Alizarin Red S images at 100x magnification showed no alterations in the presence of DSP or calcium deposits (Figure 7). In Figure 7A a complete pulp tissue disorganization was observed with an increase in loose connective tissue density. Figure 7B shows an increase in calcium deposition in the disorganized pulp tissue. However, in this image can be observed a zone of pulp tissue with normal characteristics, maintaining the integrity of the odontoblasts layer and without signs of inflammatory infiltrate (Figure 7B). To group 4, the Biodentine™ used in the extensive exposure zone was observed the presence of a slight inflammatory infiltrate with cells such as polymorphonuclear neutrophils. The pulp tissue was disorganized only in the exposure zone, with the maintenance of the odontoblast monolayer outside this area (Figure 7). H&E (Figure 7C) and staining with Alizarin Red S (Figure 7D) images at 100x magnification, in addition to corroborating the described findings, do not show alterations in the presence of DSP or calcium deposits.

On days three and seven, the inflammatory reaction decreased gradually in Group 3, but increased in Group 4.

**Figure 8:**
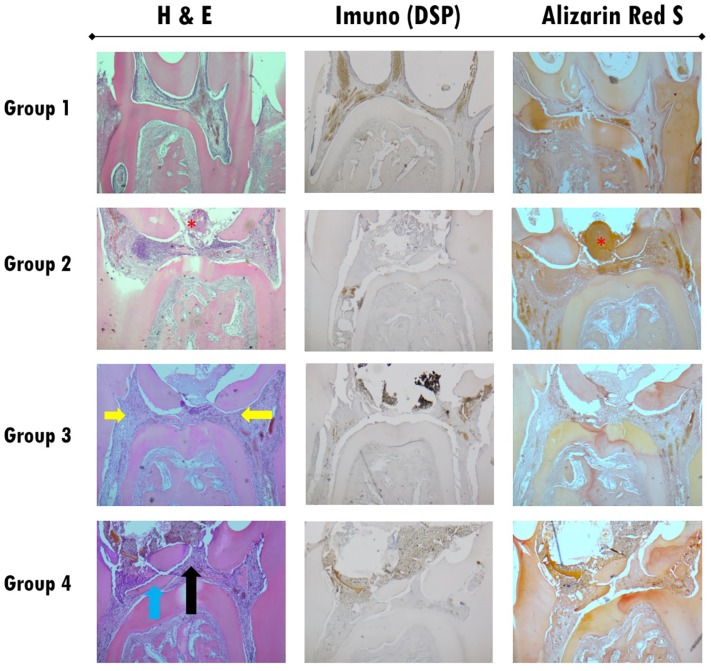
Histological sections images of all studied groups at seven days follow-up, stained with H&E (left column), with immunohistochemistry for DSP (central column) and with Alizarin Red S (right column). All images were obtained with 40x magnification. In Group 2, the pulp polyp is visible with a disorganization of the tissue inside the pulp chamber (red asterisk). In Group 3, shows a decrease in inflammatory infiltrate without mineralized tissue formation (yellow arrows). Group 4 shows an intense inflammatory infiltrate especially in the area near the exposure (black arrows) and the formation of small focuses of mineralized tissue in the exposure location (blue arrows)

**Figure 9:**
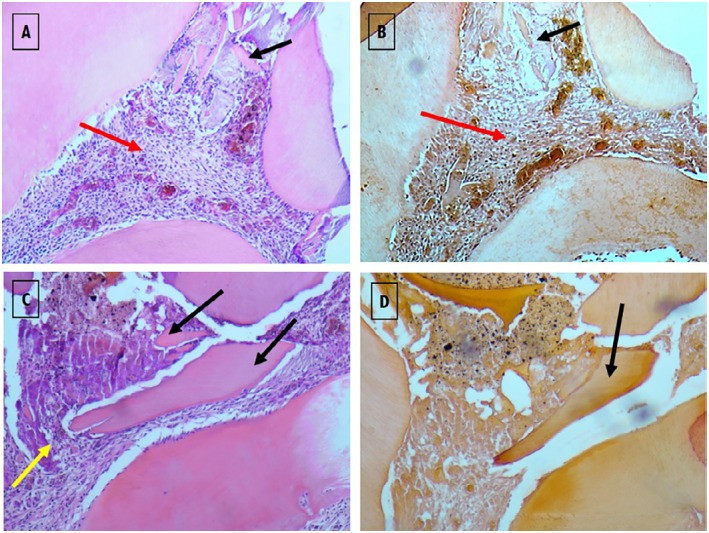
Images of histological sections in groups 3 (images A and B) and 4 (images C and D), in which therapies with WhiteProRoot^®^ MTA and Biodentine™ were performed, at 7 days of follow-up. Images A (Group 3) and C (Group 4) were stained with H&E and obtained with 100x magnification. The B (Group 3) and D (Group 4) images were stained with Alizarin Red S and obtained with 100x magnification. Black arrows – zones corresponding to mineralized tissue; red arrows – presence of slight inflammatory infiltrates and maintenance of tissue morphology; yellow arrow – intense inflammatory infiltrates in the exposure

On day seven (Figures 8 and 9), the matrix calcification could be observed, and it was more visible in the Biodentine^™^ group (Figure 8). In the treatment with WhiteProRoot^®^ MTA (Figure 8A), the presence of mild inflammatory infiltrate with neutrophil polymorphonuclear cells, tissue morphology maintenance, and presence of mineralized deposits near the exposure zone were observed, without being characteristic of a complete dentin bridge. Figure 8B shows an increase in calcium deposition in areas already identified by H&E staining. In treatment with Biodentine™ with H&E staining (Figure 8C), intense inflammatory infiltrate could be observed in the exposure zone with mineralized tissue presence. Figure 8D shows the moderate deposition of hard tissue immediately below the exposure zone and a slight deposition in other areas of the pulp tissue.

**Figure 10:**
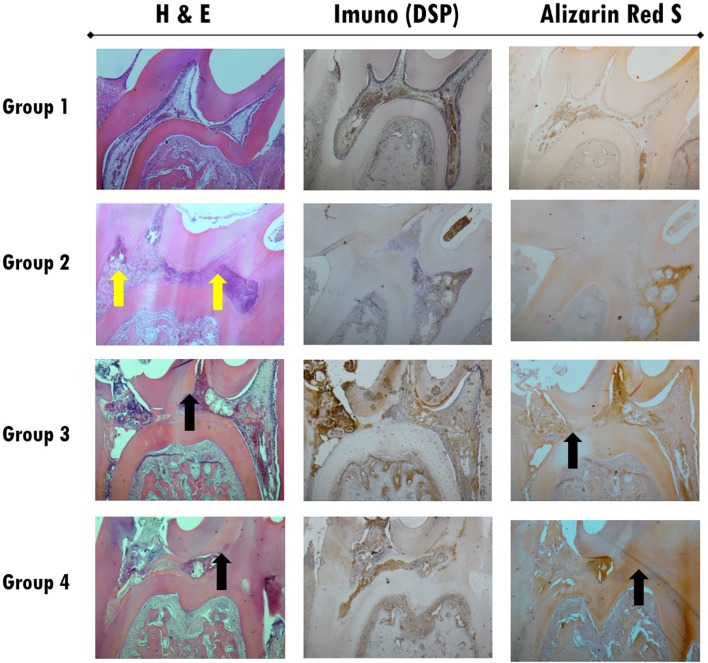
Images of histological sections of all studied groups at twenty-one days of follow-up, stained with H&E (left column), by immunohistochemistry for DSP (central column) and with Alizarin Red S (right column). All images were obtained with 40x magnification. Inflammatory reactions continue to exist in the three groups but are more evident in Group 2 with a total pulp tissue disorganization and zones of necrosis (yellow arrows). Reparative dentin formation could be observed in both Groups 3 and 4 (black arrows), and the Biodentine™ group showed more formation of reparative dentin with expansion into other dental pulp areas

**Figure 11:**
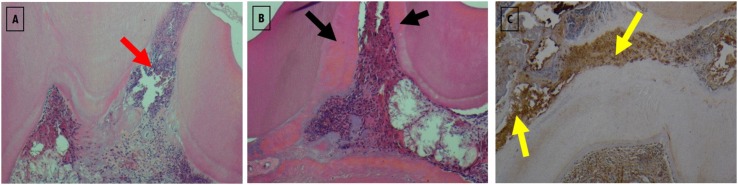
Images of histological sections of groups 2, 3 and 4 at 21 days of follow - up stained with H&E and by immunohistochemistry for DSP. The A image corresponding to group 2 (positive control) was stained with H&E and obtained with 100x magnification. Red arrow – intense inflammatory infiltrate with total disorganization of the pulp tissue. Image B corresponding to Group 3 (WhiteProRoot^®^ MTA therapy) was stained with H&E and obtained with 100x magnification. Black arrows – deposition of mineralized tissue near the exposure zone, immediately adjacent to the dentin tissue, revealing an incomplete dentin bridge. The image C corresponding to Group 4 (Biodentine™ therapy) was stained by immunohistochemistry for the DSP and obtained with 100x magnification. Yellow arrows – intense DSP marking next to the pulp exposure zone

At twenty-one days (Figures 10 and 11), inflammatory cell infiltration could be found. In the WhiteProRoot^®^ MTA group, the structure beneath the pulp tissue was normal, while in the Biodentine^™^ group, pathological calcification or inflammatory cell infiltration on pulp tissue were observed. Dentin bridges could be observed in both groups. Both the WhiteProRoot^®^ MTA and Biodentine^™^ groups showed a mild-to-moderate level of inflammatory cell infiltration and pulp tissue disorganization in all observation sites. Formation of mineralized tissue could initially be observed on day seven post operation in the Biodentine^™^ group. The Biodentine^™^ group showed much more formation of mineralized tissue at seven and twenty-one days compared with the WhiteProRoot^®^ MTA group. Alizarin Red S stain confirms the observations with hematoxylin-eosin, with mineralized tissue evidence. At twenty-one days (Figure 10), totally mineralized areas of the dental pulp were observed in Group 4. The specific odontoblastic DSP markers were mainly positively expressed in predentin and odontoblasts. The expression trend of DSP in the groups was consistent. The trend enhanced gradually from three to twenty-one days in both test groups. Generally, from seven to twenty-one days, their expression in the Biodentine^™^ group was higher than the WhiteProRoot^®^ MTA group. The observation of the images stained by immunohistochemistry revealed an increase in DSP especially near the area of the pulp exposure (Figure 11C).

The sites where WhiteProRoot^®^MTA was administered (Group 3), intense inflammatory infiltration was observed after three days of follow-up (Figures 6, 7A and 7B), with pulp tissue morphology disorganization. However, these inflammatory characteristics were present only at an early stage, which a gradual decrease in its intensity were observed as well as pulp tissue reorganization after seven days of follow-up (Figures 8, 9A and 9B). In addition, we also observed the maintenance of the peripheral monolayer of odontoblasts throughout the tissue, except in the exposure zone. After twenty-one days of follow-up, the formation of a mineralized tissue was observed next to the exposure zone, with different morphological characteristics from the original dentin tissue (Figures 10 and 11B). This formation of mineralization was not detected in other pulp tissue areas, which indicates the very localized induction of repair of the lesion through the formation of a dentin bridge.

In the Biodentine^™^ therapy group (Group 4) there were some distinct features of the treatment referred above. After three days of follow-up, the observed inflammatory infiltrate was slight, accompanied by a disorganization of the pulpal morphology only of odontoblasts peripheral layer in the exposure zone (Figures 6, 7C and 7D). These inflammatory signs increased in intensity over follow-up stages, accompanied by increased disorganization of cell morphology, which was observed after seven and twenty-one days (Figures 8, 9C, 9D and 10). The formation of mineralized tissue was observed after seven days of follow-up (Figure 9C and 9D), with the appearance of some mineralized zones near the exposure zone. At twenty-one days, several mineralized zones were observed near the exposure area and throughout the pulp tissue, called generalized pulp calcifications (Figure 10).

**Figure 12:**
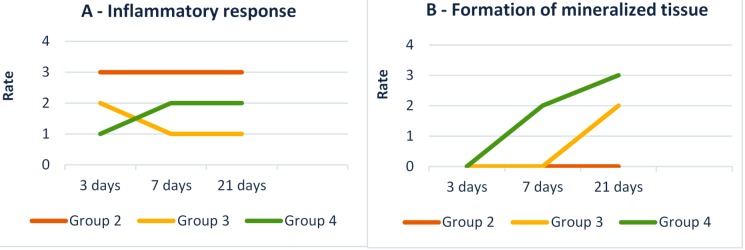
Results of histopathological assessment according to the modified version of ISO 10993 and 7405. The Y-axis corresponds to the rate of A – inflammatory response by observation of inflammatory infiltrate and disorganization of pulp tissue; and B – mineralized tissue formation by the observation of reparative dentin formation. The X-axis corresponds to the study periods

The evolution of the inflammatory response and mineralized tissue formation throughout the study stages corroborate the histological findings (Figure 12).

## Discussion

The methodology for preclinical biocompatibility assessment of materials or devices for use in Dental Medicine is determined by ISO 7405.^32^ This standard states that only non-rodent mammals such as monkeys, miniature pigs and dogs are suitable species for animal research in dental medicine.^32^^,^^33^ However, several biocompatibility studies have been published in the last years with the use of rat molar teeth, in order to assess tissue reactions after pulp tissue exposure. Rat molar teeth, including pulp tissue, can be considered anatomically, histologically, biologically, and physiologically as a miniature of human molar teeth. Thus, the essential biological reactions of pulp tissue and the interactions that occur during different healing stages in rat molar teeth are comparable to those of other mammals.^34^^–^^42^ Some authors report that rat molar teeth are a valid model to provide data on tissue reaction after pulp exposure, although they have exceptional resilience and regeneration capacity that should be considered. Thus, the use of rats can significantly reduce the number of animals currently used in research, with considerable ethical and economic advantages.^40^

As previously mentioned, the anatomical and histological similarity of rat mandibular molars to human molars was also observed in this study.^43^^,^^44^ The dentin structure that surrounds all the pulp tissue was observed, with the visualization of the dentinal tubules such as humans’ one. Several cell populations can be observed in the pulp tissue, namely fibroblasts, blood cells, nerve cells, undifferentiated cells, and odontoblasts, arranged at the periphery of the entire pulp tissue. In anatomical terms, rat's first molar is similar to human's lower molars, both in regard to the coronary structure, with three cusps, and in the root structure, with two mesial and distal roots. These characteristics were observed in the negative control group.

The positive control group was also important in this study, since it enabled the test on our experimental model. In fact, it was observed that after rat pulp tissue exposure to the external oral cavity environment, a severe inflammatory process with intense inflammatory infiltrate and a total disorganization of the cellular morphology, compatible with a necrotic tissue, is initiated. These changes are visible at all follow-up stages, always maintaining similar characteristics throughout them. Therefore, it is possible to affirm that the non-treatment of this clinical situation develops a biological process culminating in the pulp tissue necrosis.

Thus, the histological analysis of the therapies performed with the two biomaterials showed that the initial inflammatory reaction is more intense with WhiteProRoot^®^ MTA than with Biodentine^™^, and is then reversed with a decrease of these signals in the first biomaterial and an increase in the second. These intense inflammatory signs are followed proportionally by a disorganization of the pulp tissue morphology. Regarding the mineralized tissue formation, it is not observed after three days of follow-up, that any biomaterials since the induction of the formation of this type of tissue depends on several complex processes stimulation. In Biodentine^™^ therapy, this formation starts earlier but proves to be nonspecific and exaggerated. In WhiteProRoot^®^ MTA therapy, mineralized tissue formation is observed only after twenty-one days of follow-up, and in an organized and localized manner at exposure site. These results are corroborated with the molecular imaging technique by nuclear medicine. Following administration of ^99m^Tc-HMDP, a radiopharmaceutical is captured and accumulated in the bone in view of its high affinity for hydroxyapatite crystals, an integral part of the bone matrix. The bone matrix is 50% composed of inorganic matter which includes ions such as phosphate, calcium, magnesium, sodium, and citrate. Of these bone matrix components, the calcium and phosphate ions represent a large part of the bone forming inorganic component such as the hydroxyapatite crystals. Considering the results obtained were observed an increase in calcification. Calcium accumulation will not represent an increase in the radiopharmaceutical uptake between the groups, since its retention into the cell, require the presence of phosphate.

Some authors report that MTA-based cement induces pulp tissue recovery as well as reparative dentin formation superior to calcium hydroxide-based cement in the dog, monkey, and rat models.^45^ In another study, carried out with miniature pigs, the mineralized tissue formation in a dentin bridge shape was observed similarly with WhiteProRoot^®^ MTA and Biodentine™.^46^ Other authors conclude that mineralized tissue formation with Biodentine™ therapy translates into thicker and more morphologically organized dentin bridges than those using MTA-based cements.^47^^,^^48^ In other studies, reparative dentin formation has demonstrated in wells restored with Biodentine™ in miniature pigs, with a significant deposition increase when compared with calcium hydroxide-based cement.^49^

In this study, the presence of dentin sialoprotein was assessed by immunohistochemistry, showing an increase in its expression after twenty-one days of follow-up with both biomaterials. These histological findings corroborate with data obtained in the Alizarin Red S staining, proving its direct relation to mineralized tissue formation. Although the intensity of DSP marking did not reveal differences between the studied biomaterials, the observation of mineralized tissue formation showed significant differences, with a higher incidence in the treatments with Biodentine™. Other studies have concluded that Biodentine™ stimulates the same markers as ProRoot^®^MTA, but with stronger DSP labeling in dentin bridges area.^50^ Other authors have observed higher levels of DSP expression in treatments with ProRoot^®^MTA than with Biodentine^™^. However, dentin bridge formation was observed in treatments with ProRoot^®^MTA and Biodentine™ in a similar way, although with different characteristics in morphology and thickness. The authors note that these differences in dentin bridge quality may be explained by the greater disorganization of Biodentine™-induced cell morphology.^22^ These data corroborate the results of our *in vitro* study, demonstrating increased biocompatibility of WhiteProRoot^®^MTA, with less interference in cell proliferation, viability, death, and cell cycle types when compared to Biodentine™. In addition, in our study, a greater disorganization of the pulp tissue morphology can be observed in the treatment with Biodentine™.

Other materials, such as the demineralized bone matrix, have also been tested in direct pulp capping therapies in the rat model, with promising results and superior to calcium hydroxide-based cement, showing their ability to promote repair through dentin bridges.^20^

The biocompatibility of dental materials is essential to prevent inflammatory reactions appearance as well as enable tissue regeneration. The italiccutaneous implantation method is a valid methodology to determine the biocompatibility of the materials. Some authors have adopted this methodology, showing that Biodentine™ exhibits an initial inflammatory response, but this response is rapidly followed by connective tissue formation, indicating the absence of tissue irritation.^51^ In other italiccutaneous compatibility studies in rats, the authors demonstrated that the number of inflammatory cells and IL-6 was significantly higher with Biodentine™ when compared to MTA after seven and fifteen days of follow-up. However, after 60 days, a significant regression of the inflammatory reaction was observed, and both materials showed capsules with numerous fibroblasts and collagen fibers.^52^

Although in methodological terms there is a great disparity between the studies as already mentioned, there is generally a consensus regarding the induction of mineralized tissue formation with both biomaterials, ProRoot^®^ MTA and Biodentine^™^.^53^^–^^55^ The superior performance of calcium hydroxide-based cements is also consensual. Concerning the presence of an intense inflammatory reaction throughout the follow-up stages of the studies, some controversy persists. The inflammatory reaction of the pulp tissue after treatments with ProRoot^®^ MTA is mild to moderate, as previously discussed in most studies. In treatments with Biodentine™, some authors report a more intense initial inflammatory reaction with a decrease over time while others report the opposite, as found in our study. However, in no other investigation were reported the exaggerated mineralized tissue formation in treatments with Biodentine™. The histological findings related to the uncommon increase in mineralized tissue formation in treatments with this biomaterial observed in our animal study are extremely relevant for later clinical use of Biodentine™. In addition, these findings follow other *in vitro* studies conclusion, in which there was already a significant increase in the production of alkaline phosphatase, dentin sialoprotein and in the formation of calcium deposits in the treatments with Biodentine™ when compared with WhiteProRoot^®^ MTA.

This study had limitations due to difficulties in some technical procedures and histological samples processing. The difficulty of performing pulp exposures due to the animal oral cavity small size proved to be a problem. The processing of the samples, namely in the decalcification process and consequently in obtaining the consecutive histological sections was complicated. The need for consecutive histological sections to better observe and compare the different staining was difficult to obtain in some groups. The translation into the clinic is also a limitation since animal metabolism is different from human's metabolism. However, this study is a indicative of the differences between biomaterials and Biodentine™ potential calcification.

## Conclusions

We can conclude that the treatment with these biomaterials based on mineral trioxide aggregate (WhiteProRoot^®^ MTA) and tricalcium silicate (Biodentine^™^) present slight and reversible inflammatory signs in the pulp tissue, with the formation of mineralized tissue.

However, the exacerbated induction of mineralized tissue formation with the tricalcium silicate biomaterial may lead to the formation of pulp calcifications in human teeth, as already observed in the animal study, and may clinically hamper the use of this biomaterial. More randomized clinical studies or cohort studies will be needed to assess the consequences of this exaggerated increase in mineralized tissue production by tricalcium silicate.
